# Antioxidant Activity of Maillard Reaction Products and Consumer Acceptance of Nurungji Fortified with Sorghum (*Sorghum bicolor* L.)

**DOI:** 10.3390/foods15071216

**Published:** 2026-04-02

**Authors:** Woo-Ju Wee, Eun-Jung Kwak, Il-Sook Choi

**Affiliations:** 1 Department of Food and Nutrition, Wonkwang University, 460 Iksandae-ro, Iksan 54538, Republic of Korea; skwee7@naver.com; 2 Department of Food Science & Technology, Yeungnam University, 280 Daehak-ro, Gyeongsan 38541, Republic of Korea; 3 Institute for Better Living, Wonkwang University, 460 Iksandae-ro, Iksan 54538, Republic of Korea

**Keywords:** *Sorghum bicolor* L., nurungji, antioxidant properties, ferulic acids, MRP model solution

## Abstract

Sorghum *(Sorghum bicolor* L.) is an environmentally friendly crop known for its nutritional and bioactive properties. This study investigated the effects of sorghum on the antioxidant properties and consumer acceptance of nurungji, a traditional Korean snack. To understand the antioxidant contribution of ferulic acid in sorghum during non-enzymatic browning, the antioxidant activity of ferulic acid was evaluated using a sugar (glucose or fructose)–lysine Maillard reaction model system. Nurungji samples were prepared with varying sorghum blending ratios (SN0, SN25, SN50, SN75, and SN100) and heating durations (0, 1, 3, and 5 min). The total polyphenol and total flavonoid contents of nurungji increased significantly in a sorghum concentration- and heating duration-dependent manner. Antioxidant activities, including DPPH and ABTS radical scavenging activities, ferric reducing antioxidant power (FRAP), and reducing power, exhibited trends similar to those of the antioxidant components. In the isolated model system, the addition of ferulic acid significantly enhanced the antioxidant capacity of the Maillard reaction products (MRPs), with the fructose–lysine–ferulic acid solution exhibiting the highest activity. These results support the proposed mechanism that during the thermal processing of nurungji, complex polymeric phenolic compounds within the sorghum are degraded, releasing free ferulic acid that actively boosts the overall antioxidant properties of the resulting MRPs. Although the antioxidant properties of nurungji increased with higher sorghum concentration, the consumer acceptance evaluations indicated that SN0 and SN25 received significantly higher scores for overall acceptance, taste acceptance, and purchase intention (*p* < 0.05).

## 1. Introduction

Sorghum (*Sorghum bicolor* L.) is the fifth most-consumed grain worldwide. Owing to its resistance to high temperatures and drought, sorghum exhibits notable adaptability to diverse environments [1]. It also efficiently converts solar energy and requires less water than other grains, making it valuable for addressing energy and climate challenges [2,3]. In 2023–2024, global sorghum production was projected at 52.8 million tons, with the United States contributing 8.07 million tons, accounting for 14% of total production [4]. Beyond its agricultural significance, sorghum serves a broad range of industrial applications, including animal feed, biofuels, adhesives, waxes, dyes, windbreaks, and mulching [5]. Sorghum is also consumed as a food product and is used to produce bread, biscuits, starch, sugar, syrup, alcohol, beer, and malt, due to its high content of phenolic compounds such as phenolic acids, flavonoids, and tannins [6]. Among the phenolic acids contained in sorghum, the benzoic acid series (gallic acid, vanillic acid, protocatechuic acid, etc.) and the cinnamic acid series (ferulic acid, caffeic acid, para-coumaric acid, etc.) are representative phytochemical components [7]. In particular, ferulic acid is a major functional component, accounting for approximately 90% of the total phenolic acids [6,7]. Among these notable compounds, ferulic acid is primarily found bound to polysaccharides in plant cell walls and is associated with a wide range of health benefits, including antioxidant, anti-inflammatory, antimicrobial, antiallergic, anticarcinogenic, antithrombotic, antiviral, and vasodilatory effects [6,8,9,10].

Nurungji, a traditional Korean food, is the browned rice layer formed at the bottom of the rice cooker during cooking through a process of gelatinization and dextrinization [11]. When nurungji is prepared at high temperatures, it develops distinctive qualities, including a low moisture content, a crispy texture, reduced microbial growth, and minimal lipid oxidation [12,13]. Grain type and cooking methods yield different varieties of nurungji, affecting taste and texture. Early research predominantly focused on manufacturing conditions, such as cooking conditions [14], heating conditions [15,16], microwave treatment [17], stirring and ultrasonic treatment [18], and the addition of liquefying enzymes [19]. In recent years, the scope of research has expanded to evaluate the effects of various supplemental ingredients, such as purple sweet potato powder [20], *Raphanus sativus* powder [21], different rice varieties [22], barley sprout powder [23], kelp [24], and green whole grains [25].

The most prominent characteristic of nurungji stems from the Maillard reaction, which occurs during its production [26]. This amino–carbonyl reaction between amino acids, proteins, and reducing sugars typically occurs during cooking or processing. The Maillard reaction is a multi-step process, which begins with a carbonyl group and an amino group combining to form a Schiff base. The rearrangement takes place to produce an Amadori or Heyns product. Lastly, the Amadori product decomposes to form various compounds, including α-dicarbonyl compounds, methylglyoxal, and diacetyl [27,28,29]. As the reaction reaches the advanced stage, melanoidins and advanced glycation end-products (AGEs) are generated through condensation and polymerization [30,31]. Throughout these stages, various flavors and antioxidant substances are generated, known as Maillard reaction products (MRPs) [30].

Factors affecting the formation of MRPs are divided into intrinsic factors, such as food composition and structure, and extrinsic factors, such as pH, temperature, and water content [32]. MRPs have reported benefits, including enhanced food color, flavor, and taste; however, MRPs can also lead to the formation of harmful compounds, such as benzopyrene, AGEs, and acrylamide [31,32]. Regarding food safety aspects of MRPs, hydroxymethylfurfural (HMF), acrylamide, furosine, heterocyclic amines, and 3-deoxyglucosone may negatively impact health [32,33]. Meanwhile, notable reports have indicated that polyphenols may inhibit the generation of AGEs and 5-HMF [34]. Therefore, a comprehensive understanding of the factors influencing MRP formation, along with insight into both the positive and negative outcomes, is essential for future research.

This research was based on the following hypotheses. First, polyphenols in sorghum fortification would improve the antioxidant activities of nurungji under adequate heating conditions. Second, ferulic acid, the key phenolic component in sorghum, would affect antioxidant activities during MRP production. Third, an increase in concentration would positively impact consumer acceptance across sensory attributes, such as appearance, flavor, taste, and texture. The effects of sorghum fortification and heating duration on the physicochemical characteristics and antioxidant properties of nurungji were evaluated. The effect of sorghum fortification on antioxidant properties was analyzed using glucose–lysine and fructose–lysine with ferulic acid MRP model systems. Furthermore, consumer acceptance was assessed to determine optimal sorghum fortification concentrations.

## 2. Materials and Methods

### 2.1. Materials

Rice (*Oryza sativa* L.) and sorghum (*Sorghum bicolor* L.) were purchased from Iksan, South Korea, and refrigerated (C110AK, LG Electronics, Seoul, Republic of Korea). D-glucose, D-fructose, L-lysine, and trans-ferulic acid were obtained from Sigma-Aldrich (St. Louis, MO, USA) and Tokyo Chemical Industry Co., Ltd. (Tokyo, Japan) for use in the MRP model solution. Folin–Ciocalteu reagent, sodium nitrite, 2,2-diphenyl-1-picrylhydrazyl (DPPH), (±)-6-hydroxy-2,5,7,8-tetramethylchromane-2-carboxylic acid (Trolox), 2,2′-azino-bis(3-ethylbenzothiazoline-6-sulfonic acid) (ABTS), iron(III) chloride hexahydrate, potassium ferricyanide(III), Rochelle salt, and gallic acid were obtained through Sigma Aldrich (St. Louis, MO, USA) and Daejung Chemicals & Metals Co., Ltd. (Siheung-si, Republic of Korea) for antioxidant evaluation.

### 2.2. Preparation of Nurungji Fortified with Sorghum

Rice (500 g) and sorghum (500 g) were washed separately five times under tap water at a 1:2 grain-to-water ratio, with each cycle lasting 30 s at room temperature. The grains were subsequently drained using an orbital shaker (SHO-2D, DAIHAN Scientific, Daegu, Republic of Korea) at 200 rpm for 1 h. Blends of rice and sorghum (0, 25, 50, 75, and 100%) were cooked for 30 min in an electric rice cooker (SRP-H1051FI, Cuckoo, Yangsan, Republic of Korea) using filtered purified water (HF-P20S, HIFIL TECH INC, Yongin, Republic of Korea) at a 1:1.2 grain-to-water ratio.

Nurungji was prepared using a nurungji maker (BE-5200, BETHEL-COOK, Hwaseong, Republic of Korea) preheated to 210 °C. For each sample, 5 g of the cooked grains were processed for 0, 1, 3, and 5 min under controlled temperature. Individual samples were homogenized to a particle size of <500 µm using a grinder (SFM-700SS, Hanil, Wonju, Republic of Korea) and stored in a freezer (C110AK, LG Electronics, Seoul, Republic of Korea) at −18 °C for five days prior to analysis.

### 2.3. Preparation of MRP Solution

Preparation of glucose/fructose and lysine MRP solutions was performed as described by Martins and Van Boekel [35]. D-glucose and D-fructose were individually dissolved in HPLC-grade water (Honeywell Burdick & Jackson, Daejung, Republic of Korea). For samples without trans-ferulic acid (GL and FL), 1 mL of 0.1 M D-glucose or D-fructose was mixed with L-lysine at a 1:1 ratio. For samples with trans-ferulic acid (GLF and FLF), 0.2 mL of 2.5 mM trans-ferulic acid was added to each sugar–lysine mixture. Initial pH values prior to the heating process 10.11 ± 0.14, 10.11 ± 0.17, 9.92 ± 0.22, 9.88 ± 0.20 for GL, FL, GLF, and FLF, respectively. All solutions were heated in a dry block heater (ThermoMixer C, Eppendorf, Hamburg, Germany) at 100 °C for 0, 2, 4, or 6 h. The solutions were immediately cooled on ice and then stored at 4 °C for five days until analysis.

### 2.4. Physicochemical Characteristics

Moisture and ash contents were each measured using 1 g of sample in accordance with AOAC guidelines [36]. Moisture was assessed by the air-oven method at 105 °C, and ash content was assessed by the dry ash method at 550 °C. The pH was measured using 3 mL of the sample and a pH meter (S220-K, Mettler Toledo International, Inc., Seoul, Republic of Korea). For color and browning intensity analysis, samples were centrifuged at 16,000 rpm for 20 min using a Combi 524R (Hanil, Daejeon, Republic of Korea). Color measurements were assessed on 2 g of sample powder uniformly spread in a 35 × 10 mm Petri dish and using a colorimeter (CR-10 Plus, Konica Minolta Holdings, Inc., Tokyo, Japan). The colorimeter was calibrated with a white plate (L* = 97.5; a* = −0.5; b* = 3.0). Color differences (∆E*) between the control and the samples were calculated as follows:

(1) $$∆E*={\left[{\left(∆L*\right)}^{2}+{\left(∆a*\right)}^{2}+{\left(∆b*\right)}^{2}\right]}^{\frac{1}{2}}$$

Browning intensity was measured from 1 mL of the sample using a spectrophotometer (UV-1800, Shimadzu, Tokyo, Japan) at 420 nm. Reducing sugar content was measured using 3,5-Dinitrosalicylic acid (DNS). A 3 mL sample was mixed with 3 mL of 1% DNS reagent and incubated in a 90 °C water bath for 5 min. While the mixture was still warm, 1 mL of 40% Rochelle salt (potassium sodium tartrate) solution was added. After cooling to room temperature, absorbance was measured at 540 nm, and the results were calculated using a glucose standard curve (mg/g).

### 2.5. Antioxidant Component Analysis and Antioxidant Activity Assay

Prior to antioxidant analysis, the samples were first centrifuged at 4000 rpm for 5 min, then at 16,000 rpm for 20 min. Total polyphenol content (TPC) was measured using the method described by Dewanto et al. [37]. The sample (100 μL) was reacted with Folin–Ciocalteu (50 μL) reagent for 3 min, followed by the addition of 2% Na_2_CO_3_ (1 mL). The mixture was stored in the dark for 30 min, and the absorbance was measured at 750 nm using a spectrophotometer. The results were calculated using the gallic acid (mg/g) standard curve equation. Total flavonoid content (TFC) was evaluated according to a modified method by Shen et al. [38]. The sample (100 μL) was mixed with 5% NaNO_2_ (75 μL) and reacted in the dark for 5 min. The mixture was then mixed with 10% AlCl_3_·6H_2_O (150 μL) and reacted for 6 min in the dark. And lastly, 1 M NaOH (500 μL) was mixed into the mixture and stored in the dark for 20 min. Absorbance was measured at 415 nm with the spectrophotometer, and results were computed with the rutin (mg/g) standard curve equation.

DPPH radical scavenging activity was evaluated using a modified method described by Blois [39]. The DPPH reagent (0.2 mM) was adjusted with methanol to an absorbance of 1.0. The sample (100 μL) was mixed with DPPH reagent (1 mL) and incubated in the dark for 30 min. Absorbance was measured at 517 nm and calculated as a percentage relative to a control containing 100 μL of distilled water and DPPH reagent.

(2) $$DPPH\;radical\;scavenging\;\left(\%\right)=\left[\left(A_{control}-\frac{A_{sample}}{A_{control}}\right)\right]\times 100$$

ABTS^+^ radical scavenging activity was determined based on the method by Re et al. [40]. Briefly, 7 mM ABTS was mixed with 2.4 mM potassium persulfate at a 1:1 ratio and incubated in the dark to react for 12 h. The ABTS radical cation (ABTS^+^) solution was diluted with phosphate-buffered saline (PBS) to an absorbance of 0.7. The diluted ABTS^+^ solution (1 mL) was mixed with the sample (100 μL) and incubated in the dark for 30 min. Absorbance was measured at 735 nm, and results were calculated using a Trolox standard curve (mM).

The ferric reducing antioxidant power (FRAP) assay was performed according to the method of Benzie and Strain [41]. The FRAP working solution was prepared by mixing 0.2 M sodium acetate buffer (pH 3.6), 10 mM TPTZ (2,4,6-tripyridyl-S-triazine), 20 mM ferric chloride hexahydrate, and distilled water in a 10:1:1:1 volumetric ratio. The samples were incubated in a water bath at 37 °C for 30 min. The sample (100 μL) was added to the FRAP working solution (1 mL) and left in the dark for 30 min at room temperature. Color changes were then measured at 595 nm, and Trolox (mM) was used for the standard curve equation.

Reducing power was determined according to the method of Canabady-Rochelle et al. [42]. The sample (100 μL) was mixed with 200 mM sodium phosphate buffer (300 μL; pH 6.6) and 1% potassium ferricyanide (300 μL). The mixture was incubated in a 50 °C water bath for 20 min. Then, 10% trichloroacetic acid (300 μL) and 0.1% ferric chloride (100 μL) were added to the mixture and incubated for 10 min at room temperature. The absorbances were measured at 700 nm. The reducing power was expressed in the standard curve equation for Trolox (mM).

### 2.6. Consumer Acceptance Test

A total of 120 consumers aged 20 to 30 years were recruited from Iksan City, Jeonbuk State, Republic of Korea. The consumers showed varying levels of interest in nurungji: 37 consumers indicated interest, 51 indicated indifference, and 32 indicated no interest. All consumers reported that they had consumed nurungji previously. The consumer evaluation was conducted in accordance with the guidelines of the Wonkwang University Institutional Review Board. Every participant provided written informed consent and screened for food allergies prior to the consumer acceptance test.

The consumer acceptance tests were conducted in the controlled sensory evaluation booth over two 40 min sessions (10 A.M. and 3 P.M.). Prior to the test sessions, participants received instructions on the testing procedures and palate-rinsing protocols. The nurungji samples (5 g) were served at room temperature in an opaque sensory cup with a lid (7 cm × 3 cm). The cups were assigned a 3-digit random code and presented in a balanced order according to a mutually orthogonal Latin square design. Bottled water (Jeju Samdasoo, Jeju Providence Development Co., Jeju City, Republic of Korea) was provided with the samples to rinse the mouth during the evaluation.

All participants were asked to answer a brief demographic questionnaire including their age, gender, and diet. After completing a general characteristics questionnaire, participants were asked to evaluate five nurungji samples (SN0, SN25, SN50, SN75, and SN100) heated for 3 min. A nine-point hedonic scale (1 = dislike extremely, 5 = neither like nor dislike, and 9 = like extremely) was used to measure acceptances (overall, appearance, aroma, taste, and texture). A nine-point category scale (1 = very weak, 5 = neither weak nor strong, and 9 = very strong) was used to measure intensities (sweet and crunch). And a five-point Likert scale (1 = definitely would not purchase, 3 = may or may not purchase, and 5 = would definitely purchase) was used to measure the purchase intent.

### 2.7. Statistical Analysis

XLSTAT (Lurnivero, Denver, CO, USA) was used for statistical analysis. The analysis of variance (ANOVA) was performed on physicochemical characteristics and antioxidant properties. Pairwise multiple comparisons were determined via Duncan’s multiple range test with significant differences among means (*p* < 0.05). Consumer test data were analyzed using an ANOVA using Fisher’s least significant difference (LSD). Principal component analysis (PCA) was performed for the results related to both physicochemical characteristics and consumer acceptance.

## 3. Results and Discussion

### 3.1. Physicochemical Characteristics of Nurungji Fortified with Sorghum

The physicochemical characteristics of samples at varying sorghum blending ratios (0, 25, 50, 75, and 100%) and heating durations (0, 1, 3, and 5 min) are presented in Table 1.

The moisture content of the nurungji with (SN25, SN50, SN75, and SN100) and without sorghum (SN0) prior to heating (0 min) ranged from 52.02% to 57.30%, with SN75 and SN100 exhibiting the highest moisture content. The moisture content decreased most rapidly during the first 3 min of the heating process, from 24.59% to 32.95% at 1 min to 1.76% to 3.81% at 3 min. Then, the moisture content gradually declined from 3 min to 5 min (1.07–1.48%).

The crude ash content of the samples increased significantly in a sorghum concentration-dependent manner for each heating duration. The crude ash content increased in the order SN0 < SN25 < SN50 < SN75 < SN100, with SN100 ranging from 0.66 to 1.54%. These results are consistent with those of Htet et al. (2022) [43], who reported that sorghum contains a relatively higher mineral content compared to that of rice, wheat, and maize.

The L* value decreased significantly in all nurungji samples as heating duration increased. The L* value of SN100 was the lowest, and L* decreased in a sorghum concentration-dependent manner. The ΔE* values increased with increasing sorghum concentration and increasing heating duration. These results indicate that sorghum fortification increased color saturation, resulting in distinct visual differences.

The browning intensity of nurungji increased significantly with both higher sorghum concentrations and extended heating durations. SN100 exhibited the highest browning intensity, with the other groups in the order SN75 > SN50 > SN25 > SN0. This browning behavior aligns with Fatima et al. [44], who observed that baked sorghum-soy chips developed uniform browning due to the Maillard reaction in the absence of frying oil. Similarly, Jeong and Choi [45] reported that browning intensity increased in nurungji fortified with barley, attributing the change to reactions involving β-glucan compounds and the Maillard reaction.

### 3.2. Antioxidant Properties of Nurungji Fortified with Sorghum

**Figure 1:**
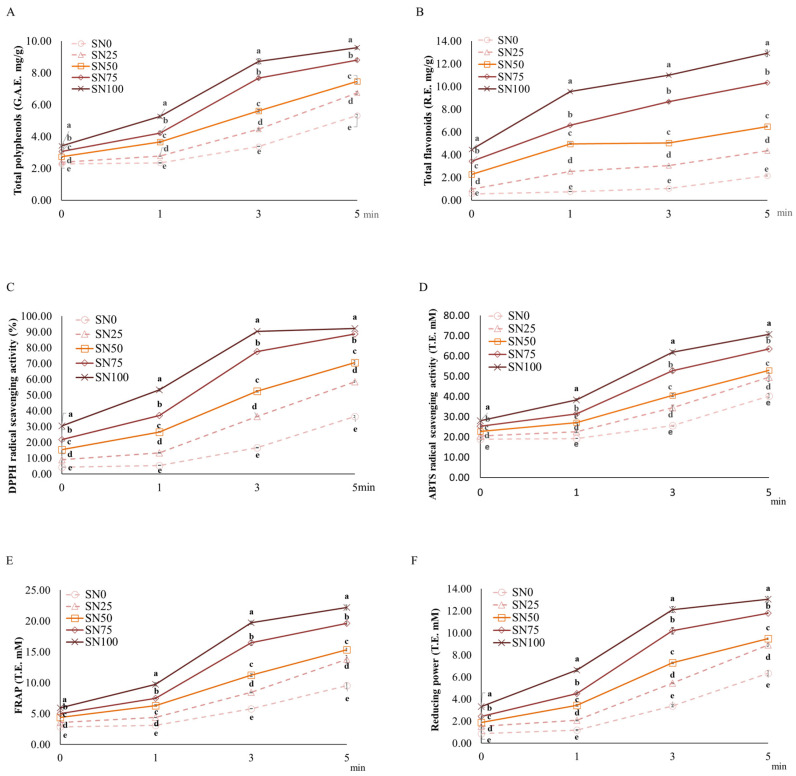
(**A**,**B**) Antioxidant contents and (**C**–**F**) antioxidant activities of the samples according to heating duration and sorghum addition ratio. Means with different superscripts (a–e) differ significantly (*p* < 0.05) across the samples at each heating time.

Figure 1 shows the antioxidant contents of nurungji fortified with sorghum. The initial TPC of the samples (2.28 to 3.40 GAE mg/g) increased with increasing sorghum concentration. TPC increased significantly with increasing heating duration, ranging from 5.32 to 9.57 GAE mg/g at 5 min (Figure 1A). The total polyphenol content of SN100 was the highest, followed by the other groups in the order SN75 > SN50 > SN25 > SN0. This concentration-dependent increase aligns with previous findings regarding the phenolic profiles of grains. Choi et al. [46] reported that red sorghum (733 mg/100 g) contains significantly higher polyphenolic compounds compared to black rice, brown rice, barley, and white rice (313 mg/100 g, 54 mg/100 g, 50 mg/100 g, and 18 mg/100 g, respectively). Tanwar et al. [47] highlighted that sorghum contains diversified phenolic compounds, including predominant phenolic acids (protocatechuic and ferulic acids) and minor phenolic acids (*p*-coumaric, syringic, vanillic, and gallic acids). Similarly, Mawouma et al. [48] identified chlorogenic acids, *p*-coumaric acids, and ferulic acids as the main hydroxycinnamic acids, as well as gallic acid, vanillic acid, and syringic acid as the main hydroxybenzoic acids, in sorghum.

TFC of the samples also increased significantly with both sorghum concentration and heating duration (Figure 1B). Consistent with the TPC results, SN100 displayed significantly higher TFC than the other samples in a concentration-dependent manner.

The initial DPPH radical scavenging activity was 4.19–30.43% and increased to 36.33–92.22% at 5 min of heating (Figure 1C) in a sorghum concentration-dependent manner. SN100 exhibited the highest scavenging activity, with the other groups in the order SN75 > SN50 > SN25 > SN0. This trend of increasing antioxidant properties with heating is consistent with Jeong and Choi (2025) [45], who observed increased TPC and TFC in barley nurungji over time. Verardo et al. [49] attributed the higher antioxidant properties of bread crust compared to dough to the formation of MRPs.

The ABTS radical scavenging activity showed a similar pattern, with SN100 displaying the highest activity (Figure 1D). The FRAP assay and the reducing power assay evaluated the reduction of ferric ions to ferrous ions under acidic pH conditions and neutral pH conditions, respectively. SN100 exhibited significantly higher FRAP and reducing power assays, followed by the other groups in the order SN75 > SN50 > SN25 > SN0 (Figure 1E,F). These increases in antioxidant activity after cooking may be related to the thermal degradation of polymeric phenolic compounds into simpler molecules, thereby increasing the free-to-bound phenolic ratio [50]. These results confirm that sorghum fortification significantly enhances the antioxidant profile of nurungji, owing to sorghum’s higher intrinsic polyphenolic content than rice [47].

### 3.3. Physicochemical Characteristics of the Model MRP Solution

Table 2 shows the model MRP solutions (GL, FL, GLF, and FLF) at different heating time intervals (0 h, 2 h, 4 h, and 6 h). The reducing sugar content decreased significantly across all samples as the heating duration increased. Notably, fructose-containing solutions exhibited lower reducing sugar content throughout the heating period compared to their counterpart glucose-containing solutions (FL < GL and FLF < GLF). This finding is consistent with Hosry et al. [51], who reported that fructose is more reactive toward amino acids than glucose, leading to a higher rate of MR and MRP formation. Theng et al. [52] explained that open-chain forms of fructose, compared to glucose, allow the sugar to react more readily with amino acids and form an amino–glucose complex. Similarly, Jeong and Choi [45] reported that fructose-derived MRPs with and without β-glucan had higher decreases in reducing sugar content than glucose-derived MRPs with and without β-glucan, a trend that aligns with the results reported in this study.

The pH of GL and GLF decreased significantly from 9.4 to 8.5 as heating duration increased, and FL and FLF decreased from pH 9.9 to pH 8.2. According to Liu et al. [53], while high pH provided an adequate condition for the molecular rearrangement of sugars, pH typically decreases during the MR as degradation compounds accelerate the formation of organic acids. Bolchini et al. [54] confirmed that under controlled conditions at 90 °C, the formation of key MRPs including acetic acid, formic acid, and melanoidins was observed over time.

The L* value of the MRP solutions decreased significantly over the first 2 h as the heating duration increased. The L* value significantly decreased in GL and FL with increasing heating duration up to 4 h, after which no significant changes were observed. The a* value significantly decreased until 4 h of heating was reached, except for FL, which showed no change past 2 h, whereas the b* value increased significantly in all samples with increasing heating duration. FL and FLF had higher b* values than those of GL and GLF once heated. These findings are consistent with Sun et al. [55], who observed that fructose-derived MRPs were characterized by a lower L* value but higher a* and b* values compared to glucose-derived MRPs. The browning intensity of the FLF was significantly higher during the heating period than the other samples, followed by FL > GLF > GL. A study by Hwang et al. [56] attributed the higher content of browning substances in the fructose-derived MRPs to higher antioxidant activity compared to glucose-derived MRPs. These results are consistent with those of Jeong and Choi [44], who reported that the browning intensity for the fructose-derived MRPs was significantly higher than that of the glucose-derived MRPs, regardless of the presence of β-glucan.

### 3.4. Antioxidant Activities of MRP Solution

**Figure 2:**
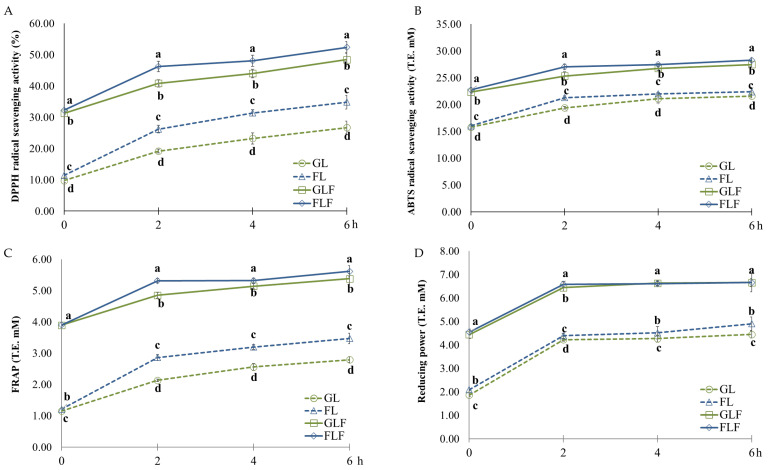
(**A**–**D**) Antioxidant activities of model MRP solutions. Means with different superscripts (a–d) differ significantly (*p* < 0.05) across the samples at each heating time.

Figure 2 shows the antioxidant activities of the glucose–lysine and fructose–lysine MRP model systems, with and without ferulic acid. The group with ferulic acid (GLF and FLF) exhibited significantly higher DPPH radical scavenging activities than the groups without ferulic acid (GL and FL) (Figure 2A). The antioxidant activity of all MRP solutions increased rapidly during the first 2 h of heating, followed by a gradual increase. The DPPH radical scavenging activity ranged from 9.74 to 26.70% in GL, 11.40 to 34.85% in FL, 31.26 to 48.45% in GLF, and 32.24 to 52.37% in FLF. The FLF showed a significantly higher DPPH radical scavenging activity, followed by GLF > FL > GL. These results align with those of Jeong and Choi [44], who reported that the DPPH radical scavenging activity of fructose–lysine (with and without β-glucan) was higher than that of glucose–lysine counterparts. According to Hosry et al. [50], fructose is more reactive toward amino acids than glucose, leading to a higher rate of MR reaction and MRP formation. Silván et al. [57] showed that Heyns rearrangement products and melanoidin formation were inhibited in the protein–fructose MRP model system with ferulic acid. In the antioxidant study of MRPs based on fructose–lysine and ribose–lysine model systems [32], moderate temperatures (60 to 80 °C) resulted in increased antioxidant activity, whereas at a higher temperature (121 °C), antioxidant activity decreased.

The ABTS radical scavenging activity in the ferulic acid-fortified group (GLF and FLF) was significantly higher than that in the ferulic acid-free groups (GL and FL) (Figure 2B). FLF exhibited the highest ABTS radical scavenging activity, followed by GLF, FL, and GL. The ABTS radicals are generated by reacting ABTS with a strong oxidizing agent, such as potassium sulfate, and are widely used to study the antioxidant activities of MRPs in various food and model samples [54]. The FRAP of the MRP solutions increased significantly with heating duration. The groups fortified with ferulic acid (GLF and FLF) showed significantly higher FRAP values compared to the ferulic acid-free control groups (GL and FL) (Figure 2C). These findings are consistent with Kim and Lee [58], who observed that fructose–lysine MRP solutions exhibited higher FRAP values than glucose–lysine MRP solutions upon heating. Furthermore, the reducing power, a common metric for assessing the antioxidant activity of MPRs, increased rapidly up to 2 h of heating and continued to rise gradually as the reaction proceeded. These results suggest that ferulic acid significantly enhances the antioxidant capacity of MRPs, with activity levels exhibiting a time-dependent increase.

### 3.5. Consumer Acceptance of Sorghum Nurungji

**Figure 3:**
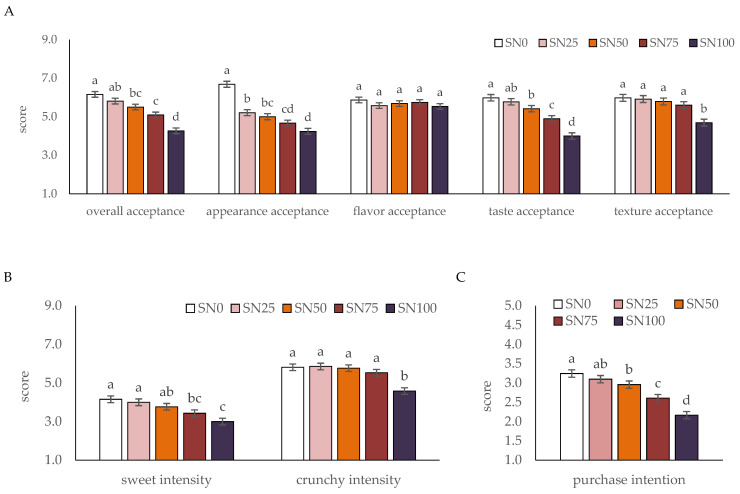
(**A**) Consumer acceptance, (**B**) attribute intensity, and (**C**) purchase intention of nurungji fortified with varying sorghum ratios. Mean values with different letters are significantly different at *p* < 0.05.

A consumer acceptance test was conducted to determine the optimal sorghum fortification level for nurungji from consumer perspectives. Figure 3 illustrates the consumer acceptance test results. Overall acceptance scores of SN0 and SN25 were high with no significant difference, and the scores decreased as the sorghum concentration increased. Appearance acceptance was significantly highest for SN0 and decreased in a sorghum concentration-dependent manner. Flavor acceptance did not differ significantly among the samples, indicating that the addition of sorghum did not negatively impact the general flavor profile perceived by consumers.

Taste acceptance and sweet intensity followed a similar trend to that of overall acceptance. SN0 and SN25 exhibited high acceptance scores with no significant difference between them; however, acceptance decreased significantly once the sorghum concentration exceeded 50% compared to SN0. These data indicate that 25% fortification was significantly more acceptable by the consumers in terms of taste acceptance than higher concentrations. Texture acceptance and crunch intensity did not differ significantly among SN0, SN25, SN50, and SN75, whereas SN100 had significantly lower scores. Purchase intention mirrored the trends observed for overall acceptance and taste acceptance, with SN0 and SN25 receiving the highest scores. These findings indicate that while antioxidant properties increased in a sorghum concentration-dependent manner, consumer acceptance favors a moderate addition level below 25%. The decline in acceptance at higher concentrations may be attributed to the physicochemical changes and sensory attributes of sorghum. Adzqia et al. [59] reported that due to phenolic compounds and tannins in sorghum, increasing the sorghum flour content in gluten-free bread resulted in increased bitterness and decreased cohesiveness, chewiness, and moisture content.

**Figure 4:**
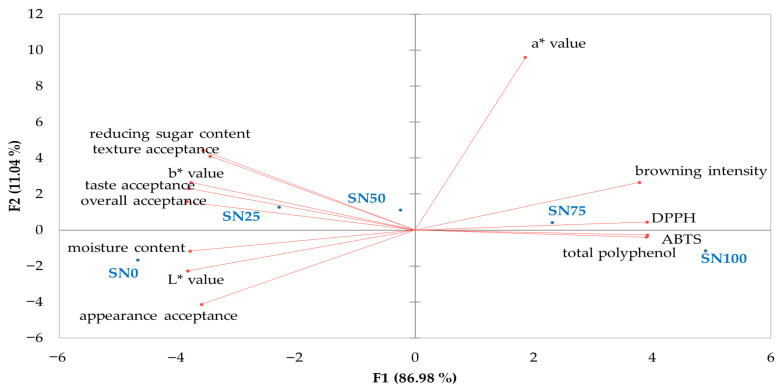
Two-dimensional PCA biplot of physicochemical characteristics, antioxidant components, antioxidant activities, and consumer acceptance (*n* = 120) of nurungji fortified with sorghum.

The results of the principal component analysis (PCA) are presented in Figure 4. The analysis shows that about 87% of the total variation is explained by the first principal component, and 11% by the first two principal components. Antioxidant components and antioxidant activities are clustered in the positive PC2 region, while consumer acceptances are found on the negative side, indicating a clear separation by analytical instrument methods. The control group (SN0) was characterized by high moisture content and L* values, which are positioned in the negative quadrants of both PC1 and PC2. In contrast, the sample fortified with 25% sorghum (SN25) was positioned in the negative PC1 and positive PC2 region, showing a strong correlation with sensory acceptance attributes. Therefore, sorghum fortification in nurungji is characterized by its impacts on moisture content, sensory acceptance, and antioxidant properties. While the 25% sorghum-fortified nurungji was highly associated with favorable sensory characteristics, fortification levels exceeding 50% showed a strong correlation between browning intensity and enhanced antioxidant properties.

## 4. Conclusions

In this study, a food matrix of sorghum-fortified nurungji and an isolated model MRP system were evaluated to understand the concentration- and time-dependent antioxidant benefits of sorghum fortification and thermal processing. Overall, the antioxidant activity of nurungji increased as both the sorghum fortification concentration and the heating duration increased. Sorghum-fortified nurungji exhibited increases in browning intensity, total polyphenol and flavonoid contents, and antioxidant activities. The increase in antioxidant activity may be attributed to greater amounts of antioxidant components, such as total polyphenols and flavonoids, that are degraded and released from the matrix during thermal treatment and Maillard reactions.

In the model MRP system, the addition of ferulic acid, a major phenolic compound in sorghum, enhanced all measured antioxidant activities, notably DPPH, ABTS, FRAP, and reducing power, which were significantly higher with MRPs containing ferulic acid. While these results suggest that ferulic acid from sorghum played a significant role in the antioxidant properties of non-enzymatic browning reaction products, it must be noted that the behavior of an isolated sugar–amino acid–ferulic acid solution was extrapolated to a complex food matrix in this study. And the direct interactions of ferulic acid within the complex carbohydrate, protein, and competing phenolic environment of nurungji should be researched.

Despite the concentration-dependent increase in antioxidant properties, the accepted level of sorghum fortification for consumer acceptance was 25% or less. And the overall acceptance and purchase intention scores were only moderate, even at these levels. This indicates that 25% sorghum fortification is tolerated, but it does not substantially drive consumer appeal compared to the unfortified control. Lastly, the correlation between sensory evaluation and flavor profile analysis was not examined in this study and should be addressed in future research.

In conclusion, while sorghum fortification positively affected the antioxidant properties of nurungji, consumer acceptance data favors a moderate sorghum concentration below 25%. Future research should focus on verifying the chemical interactions within the complex food matrix, optimizing processing methods to improve the sensory profile of sorghum, and conducting broader consumer studies to support the development of viable, health-promoting traditional snacks.

## Figures and Tables

**Table 1:** Physicochemical characteristics of nurungji fortified with sorghum at heating duration and addition ratio of sorghum.

	Heating	SN0	SN25	SN50	SN75	SN100
	(min)					
Moisture (%)	0	52.02 ± 0.26 ^d,A^	54.88 ± 0.23 ^c,A^	56.07 ± 0.09 ^b,A^	56.99 ± 0.11 ^a,A^	57.30 ± 0.08 ^a,A^
	1	32.95 ± 0.20 ^a,B^	29.58 ± 0.36 ^b,B^	25.80 ± 0.36 ^d,B^	27.78 ± 0.14 ^c,B^	24.59 ± 0.10 ^e,B^
	3	3.81 ± 0.02 ^a,C^	3.39 ± 0.04 ^b,C^	2.45 ± 0.02 ^c,C^	1.93 ± 0.02 ^d,C^	1.76 ± 0.05 ^e,C^
	5	1.15 ± 0.04 ^c,D^	1.45 ± 0.03 ^a,D^	1.07 ± 0.00 ^d,D^	1.32 ± 0.02 ^b,D^	1.48 ± 0.01 ^a,D^
Ash (%)	0	0.20 ± 0.02 ^d,C^	0.38 ± 0.02 ^c,D^	0.48 ± 0.01 ^b,C^	0.67 ± 0.02 ^a,C^	0.66 ± 0.01 ^a,C^
	1	0.30 ± 0.03 ^e,A^	0.48 ± 0.01 ^d,C^	0.73 ± 0.00 ^c,B^	1.07 ± 0.01 ^b,B^	1.19 ± 0.03 ^a,B^
	3	0.30 ± 0.01 ^e,AB^	0.67 ± 0.05 ^d,A^	0.83 ± 0.02 ^c,A^	1.35 ± 0.02 ^b,A^	1.54 ± 0.02 ^a,A^
	5	0.26 ± 0.02 ^e,B^	0.61 ± 0.01 ^d,B^	0.82 ± 0.02 ^c,A^	1.35 ± 0.02 ^b,A^	1.52 ± 0.01 ^a,A^
pH	0	4.53 ± 0.03 ^e,C^	5.80 ± 0.03 ^d,A^	6.36 ± 0.01 ^c,A^	6.69 ± 0.03 ^b,A^	6.83 ± 0.04 ^a,A^
	1	4.62 ± 0.00 ^e,B^	5.73 ± 0.01 ^d,B^	6.18 ± 0.02 ^c,B^	6.54 ± 0.01 ^b,B^	6.69 ± 0.03 ^a,B^
	3	4.54 ± 0.02 ^e,C^	5.49 ± 0.01 ^d,C^	5.94 ± 0.02 ^c,C^	6.14 ± 0.02 ^b,C^	6.21 ± 0.01 ^a,C^
	5	4.70 ± 0.01 ^e,A^	5.52 ± 0.01 ^d,C^	5.93 ± 0.02 ^c,C^	6.53 ± 0.02 ^b,C^	6.21 ± 0.02 ^a,C^
Reducing sugar (mg/g)	0	4.18 ± 0.02 ^b,D^	4.13 ± 0.01 ^c,D^	4.08 ± 0.00 ^d,D^	4.23 ± 0.01 ^a,D^	4.12 ± 0.02 ^c,D^
	1	6.05 ± 0.02 ^d,C^	7.44 ± 0.01 ^d,B^	8.01 ± 0.02 ^b,A^	8.52 ± 0.03 ^c,A^	8.51 ± 0.01 ^a,A^
	3	7.88 ± 0.03 ^d,A^	8.31 ± 0.02 ^b,A^	7.55 ± 0.02 ^a,B^	6.83 ± 0.02 ^c,B^	5.76 ± 0.02 ^b,B^
	5	6.39 ± 0.01 ^c,B^	5.89 ± 1.77 ^c,C^	4.94 ± 0.01 ^b,C^	5.24 ± 0.02 ^b,C^	4.72 ± 0.01 ^a,C^
L* value	0	72.73 ± 1.39 ^a,A^	62.47 ± 0.15 ^b,A^	56.93 ± 0.25 ^c,A^	52.10 ± 0.35 ^d,A^	50.40 ± 0.10 ^e,A^
	1	71.80 ± 0.70 ^a,A^	61.67 ± 0.12 ^b,B^	53.43 ± 0.35 ^c,B^	48.77 ± 0.15 ^d,B^	43.80 ± 0.17 ^e,B^
	3	62.90 ± 0.10 ^a,B^	52.53 ± 0.25 ^b,C^	46.40 ± 0.10 ^c,C^	40.27 ± 0.06 ^d,C^	36.83 ± 0.06 ^e,C^
	5	50.93 ± 1.46 ^a,C^	44.33 ± 0.06 ^b,D^	40.43 ± 0.25 ^c,D^	36.37 ± 0.15 ^d,D^	34.27 ± 0.06 ^e,D^
a* value	0	−2.10 ± 0.00 ^e,C^	6.30 ± 0.00 ^d,C^	7.97 ± 0.06 ^c,A^	8.40 ± 0.10 ^b,A^	8.60 ± 0.17 ^a,A^
	1	−2.60 ± 0.00 ^d,D^	5.13 ± 0.15 ^c,D^	7.33 ± 0.06 ^b,B^	7.40 ± 0.10 ^b,B^	8.00 ± 0.10 ^a,B^
	3	3.23 ± 0.21 ^e,B^	7.50 ± 0.10 ^b,A^	7.87 ± 0.06 ^a,A^	7.03 ± 0.06 ^c,C^	6.37 ± 0.06 ^d,C^
	5	6.17 ± 0.47 ^b,A^	7.00 ± 0.10 ^a,B^	6.67 ± 0.15 ^a,C^	5.33 ± 0.15 ^c,D^	4.60 ± 0.10 ^d,D^
b* value	0	−1.37 ± 0.47 ^c,C^	7.63 ± 0.12 ^b,C^	8.43 ± 0.06 ^a,B^	8.50 ± 0.00 ^a,A^	8.23 ± 0.15 ^a,A^
	1	−2.27 ± 0.35 ^c,D^	5.97 ± 0.06 ^b,D^	7.00 ± 0.20 ^a,D^	6.77 ± 0.06 ^a,C^	7.00 ± 0.10 ^a,B^
	3	10.93 ± 0.23 ^b,B^	11.17 ± 0.06 ^a,A^	9.53 ± 0.06 ^c,A^	7.57 ± 0.06 ^d,B^	6.13 ± 0.06 ^e,C^
	5	13.20 ± 0.40 ^a,A^	9.80 ± 0.06 ^b,B^	7.60 ± 0.35 ^c,C^	5.17 ± 0.15 ^d,D^	3.93 ± 0.15 ^e,D^
∆E*	0	1.08 ± 0.61 ^e,C^	16.03 ± 0.12 ^d,C^	21.14 ± 0.23 ^c,D^	25.17 ± 0.32 ^b,D^	26.56 ± 0.20 ^a,D^
	1	1.43 ± 0.66 ^e,C^	15.12 ± 0.12 ^d,D^	23.05 ± 0.20 ^c,C^	27.03 ± 0.13 ^b,C^	31.77 ± 0.11 ^a,C^
	3	16.63 ± 0.16 ^e,B^	25.23 ± 0.20 ^d,B^	29.93 ± 0.08^c,B^	35.01 ± 0.02 ^b,B^	38.00 ± 0.05 ^a,B^
	5	27.07 ± 1.12 ^e,A^	31.74 ± 0.04 ^d,A^	34.73 ± 0.19 ^c,A^	38.22 ± 0.14 ^b,A^	40.14 ± 0.04 ^a,A^
Browning intensity	0	0.04 ± 0.01 ^e,D^	0.06 ± 0.01 ^d,D^	0.12 ± 0.01 ^c,D^	0.15 ± 0.00 ^b,D^	0.19 ± 0.01 ^a,D^
	1	0.10 ± 0.01 ^e,C^	0.37 ± 0.01 ^d,C^	0.58 ± 0.01 ^c,C^	0.74 ± 0.01 ^b,C^	0.86 ± 0.00 ^a,C^
	3	0.16 ± 0.00 ^e,B^	0.49 ± 0.01 ^d,B^	0.59 ± 0.01 ^c,B^	0.84 ± 0.01 ^b,B^	0.88 ± 0.02 ^a,B^
	5	0.34 ± 0.01 ^e,A^	0.63 ± 0.01 ^d,A^	0.83 ± 0.01 ^c,A^	1.25 ± 0.02 ^b,A^	1.29 ± 0.02 ^a,A^

Significant differences (*p* < 0.05) using Duncan’s multiple range test within the same row are denoted by the superscripts (a–e) and those within the same column are denoted by the superscripts (A–D).

**Table 2:** Physicochemical characteristics of the model MRP solutions prepared with GL, FL, GLF, and FLF from 0 to 6 h of heating duration.

	Heating Time (h)	GL	FL	GLF	FLF
Reducing sugar (mg/g)	0	73.62 ± 0.74 ^b,A^	74.88 ± 1.35 ^a,A^	74.79 ± 1.78 ^ab,A^	71.29 ± 0.35 ^c,A^
	2	63.76 ± 0.80 ^a,B^	57.69 ± 1.30 ^c,B^	61.88 ± 1.59 ^b,B^	55.58 ± 1.77 ^d,B^
	4	56.17 ± 1.46 ^a,C^	51.33 ± 1.46 ^c,C^	53.87 ± 1.61 ^b,C^	49.77 ± 1.24 ^d,C^
	6	51.39 ± 0.94 ^a,D^	48.28 ± 1.32 ^bc,D^	49.58 ± 1.51 ^b,D^	47.96 ± 1.39 ^c,D^
pH	0	10.11 ± 0.14 ^a,A^	10.11 ± 0.17 ^a,A^	9.92 ± 0.22 ^b,A^	9.88 ± 0.20 ^b,A^
	2	9.47 ± 0.05 ^a,B^	9.04 ± 0.12 ^c,B^	9.31 ± 0.07 ^b,B^	9.11 ± 0.06 ^c,B^
	4	8.97 ± 0.07 ^a,C^	8.66 ± 0.11 ^c,C^	8.84 ± 0.08 ^b,C^	8.61 ± 0.09 ^c,C^
	6	8.58 ± 0.08 ^a,D^	8.23 ± 0.06 ^b,D^	8.54 ± 0.12 ^a,D^	8.15 ± 0.19 ^b,D^
L* value	0	58.69 ± 0.20 ^a,A^	58.50 ± 0.14 ^b,A^	58.31 ± 0.08 ^c,A^	58.33 ± 0.17 ^c,A^
	2	58.25 ± 0.14 ^a,B^	57.86 ± 0.19 ^c,B^	58.03 ± 0.12 ^b,B^	57.78 ± 0.17 ^c,B^
	4	57.96 ± 0.09 ^a,C^	57.48 ± 0.31 ^b,C^	57.88 ± 0.07 ^a,C^	57.78 ± 0.17 ^a,B^
	6	57.84 ± 0.05 ^a,C^	57.50 ± 0.08 ^c,C^	57.74 ± 0.13 ^ab,D^	57.61 ± 0.26 ^bc,B^
a* value	0	−1.30 ± 0.00 ^a,A^	−1.30 ± 0.00 ^a,A^	−1.30 ± 0.00 ^a,A^	−1.30 ± 0.00 ^a,A^
	2	−1.45 ± 0.05 ^a,B^	−1.51 ± 0.04 ^b,B^	−1.43 ± 0.05 ^a,B^	−1.43 ± 0.05 ^a,B^
	4	−1.51 ± 0.04 ^a,C^	−1.50 ± 0.00 ^a,B^	−1.50 ± 0.00 ^a,C^	−1.50 ± 0.00 ^a,C^
	6	−1.58 ± 0.05 ^b,D^	−1.49 ± 0.08 ^a,B^	−1.50 ± 0.00 ^a,C^	−1.50 ± 0.00 ^a,C^
b* value	0	−1.45 ± 0.16 ^a,D^	−1.48 ± 0.05 ^a,D^	−1.41 ± 0.15 ^a,D^	−1.39 ± 0.06 ^a,D^
	2	−0.18 ± 0.09 ^b,C^	0.79 ± 0.14 ^a,C^	−0.06 ± 0.09 ^b,C^	0.83 ± 0.18 ^a,C^
	4	0.75 ± 0.19 ^b,B^	1.43 ± 0.34 ^a,B^	0.94 ± 0.14 ^b,B^	1.41 ± 0.21 ^a,B^
	6	1.29 ± 0.29 ^b,A^	1.98 ± 0.39 ^a,A^	1.59 ± 0.34 ^b,A^	2.21 ± 0.25 ^a,A^
∆E*	0	0.47 ± 0.02 ^a,D^	0.36 ± 0.07 ^ab,D^	0.25 ± 0.04 ^b,D^	0.24 ± 0.01 ^b,D^
	2	1.05 ± 0.08 ^c,C^	2.06 ± 0.10 ^a,C^	1.18 ± 0.07 ^b,C^	2.10 ± 0.17 ^a,C^
	4	1.99 ± 0.17 ^b,B^	2.77 ± 0.35 ^a,B^	2.19 ± 0.13 ^b,B^	2.68 ± 0.21 ^a,B^
	6	2.55 ± 0.27 ^b,A^	3.28 ± 0.37 ^a,A^	2.85 ± 0.33 ^b,A^	3.49 ± 0.26 ^a,A^
Browning intensity	0	0.00 ± 0.00 ^b,A^	0.00 ± 0.00 ^b,D^	0.01 ± 0.00 ^a,D^	0.01 ± 0.00 ^a,D^
	2	0.10 ± 0.01 ^c,B^	0.15 ± 0.01 ^b,C^	0.11 ± 0.01 ^c,C^	0.16 ± 0.02 ^a,C^
	4	0.15 ± 0.02 ^b,C^	0.18 ± 0.01 ^a,B^	0.17 ± 0.03 ^ab,B^	0.18 ± 0.02 ^a,B^
	6	0.16 ± 0.02 ^b,D^	0.20 ± 0.03 ^a,A^	0.20 ± 0.03 ^a,A^	0.22 ± 0.02 ^a,A^

Significant differences (*p* < 0.05) using Duncan’s multiple range test within the same row are denoted by the superscripts (a–d) and those within the same column are denoted by the superscripts (A–D).

## Data Availability

The original contributions presented in this study are included in the article. Further inquiries can be directed to the corresponding authors.

## Abbreviations

The following abbreviations are used in this manuscript:

*a**	Red/green value
ABTS	2,2′—azino-bis(3-ethylbenzothiazoline-6-sulfonic acid)
AGEs	Advanced glycation end-products
ANOVA	Analysis of variance
*b**	Yellow/blue value
DNS	3,5-dinitrosalicylic acid
DPPH	2.2-diphenyl-1-picrylhydrazyl
Δ*E**	Color difference
FL	Fructose–lysine
FLF	Fructose–lysine with ferulic acid
FRAP	Ferric reducing antioxidant power
GL	Glucose–lysine
GLF	Glucose–lysine with ferulic acid
HMF	Hydroxymethylfurfural
*L**	Lightness
LSD	Least significant difference
MRPs	Maillard reaction products
TFC	Total flavonoid content
TPC	Total polyphenol content
